# Assessment of local wood species used for the manufacture of cookware and the perception of chemical benefits and chemical hazards associated with their use in Kumasi, Ghana

**DOI:** 10.1186/1746-4269-8-46

**Published:** 2012-12-18

**Authors:** John Kenneth Mensah, Evans Adei, Dina Adei, Gwendolyn Owusu Ansah

**Affiliations:** 1 Department of Chemistry, Kwame Nkrumah University of Science and Technology, Kumasi, Ghana; 2 Department of Planning, Kwame Nkrumah University of Science and Technology, Kumasi, Ghana

**Keywords:** Wood species, Food contact, Phytochemicals, Chemical hazard, Chemical benefit

## Abstract

**Background:**

Historical proven wood species have no reported adverse health effect associated with its past use. Different historical proven species have traditionally been used to manufacture different wooden food contact items. This study uses survey questionnaires to assess suppliers’, manufacturers’, retailers’ and consumers’ (end-users’) preferences for specific wood species, to examine the considerations that inform these preferences and to investigate the extent of awareness of the chemical benefits and chemical hazards associated with wooden food contact material use.

**Methods:**

Through the combined use of a cross sectional approach and a case study design, 25 suppliers, 25 manufacturers, 25 retailers and 125 consumers (end-users) of wooden food contact materials in four suburbs in Kumasi Metropolitan Area (Anloga junction, Ahinsan Bus Stop, Ahwia-Pankrono and Race Course) and Ashanti Akyim Agogo in the Ashanti Akyim North District of the Ashanti Region were administered with closed ended questionnaires. The questionnaires were prepared in English, but local language, Twi, was used to translate and communicate the content of the questionnaire where necessary.

**Results:**

Suppliers’, manufacturers’ and retailers’ preferences for specific wood species for most wooden cookware differed from that of consumers (end-users). But all respondent groups failed to indicate any awareness of chemical benefits or chemical hazards associated with either the choice of specific wood species for specific wooden cookware or with the general use of wooden food contact materials. The lack of appreciation of chemical benefits or hazards associated with active principles of wooden cookware led to heavy reliance of consumers (end-users) on the wood density, price, attractive grain pattern and colour or on the judgement of retailers in their choice of specific species for a wooden cookware.

**Conclusion:**

This study contributes some practical suggestions to guide national policy development on improvement in quality of available wooden food contact materials in Ghana.

## Background

Wooden food contact materials serve important functions in every aspect of food preparations and, collectively, constitute the most frequently utilized cookwares in the Ghanaian home. Ghana has a rich variety of trees whose wood possess unique structural, physical and mechanical properties that allows for the manufacture of different wood based food contact materials including mortar, pestle, grinding bowl, grinding pestle, roller, chopping board, banku ladle and wooden spoon [1]. Banku is a cornmeal prepared through repeated stirring and kneading of boiling fermented corn-dough slurry with a large wooden spoon or ladle. Most available indigenous wood species are, however, phytochemical-rich [2-7] and its contact with food surfaces, however brief, mediate the transfer of chemical substances from wood to food and vice versa [8-10]. Such chemical transfers are particularly pertinent to the Ghanaian setting, where carbohydrate-rich food including maize, cassava, yams, cocoyams and plantains are processed for considerable duration via repeated kneading and/or pounding with one or more wooden cookware. The type and the dose of chemical constituents transferred from wood to food are likely species-specific [9,11]. Formal recognition of such chemical transfers involving both beneficial and toxic wood phytochemicals has been slow but growing steadily in recent times [12-17]. Wood phytoconstituent migrants may elicit a wide range of beneficial and/or deleterious physiological responses in humans even at very low doses [18,19]. And because of the potentially wide exposure of the general population, including pregnant women and children, to large classes of wood phytoconstituents with unknown bioactivities and uncertain toxicology on a regular basis, wooden food contact materials have become a significant public health importance [17]. Thus, which wood species to use for the manufacture of specific food contact item and the reasons for the choice of that species remain issues of current health importance.

Some wood species are valued for the curative effects of its phytoconstituents or extractives in ethnomedicinal practices [20]. And some others are prized for the flavor imparted by its extractives to smoked meat and smoked fish [21]. But other species have no value for food contact purposes partly due to their intrinsic physical and mechanical liabilities and/or to the toxico-bioactive properties of its phytoconstituents [22-28]. Traditionally, historical knowledge has guided the continued use of specific indigenous species of wood for food contact purposes. But among the myriad casualties of tropical deforestation are the rapid losses of some historical proven species such as Odum (Chlorophora Excels), Mahogany (Khaya senegalensis) and Sapele (Entandrophragma cylindricum). Such losses have probably led to the increasing use of species in plentiful supply such as Nyamedua (Alstonia boonei), Teak (Tectona grandis) and Kyere (Pterygota macrocarpa) for food contact purposes”.

Most species that are new entrants into wooden cookware have scant historical records, unknown phytoconstituent bioactivity and uncertain chemical safety and their use for food contact purposes have potential implications for consumer food safety. In all cases, the reasons for manufacturers’ choice of the species are yet to be clearly identified. However, there is suggestive evidence that wood species are chosen by manufacturers with little or no cognizance to the chemical benefits or to the toxicological suitability for food contact uses and that end users choice of manufactured products largely influenced by species’ physical and aesthetic properties also supplants concerns over food quality and food safety [29]. However, whether this is true of all types of wooden food contact materials is far from clear. There is also mounting, but limited, evidence to suggest that availability of species is the most important influence on choice of wood for the manufacture of a food contact material [30]. But no study has provided empirical evidence on the prevalence of such food contact suitability determinant.

A comprehensive assessment of factors that determine wood species suitability for specific food contact uses was done using a survey research on a representative community sample of manufacturers, suppliers, retailers and consumers (end-users) of wooden food contact materials. Critical questions that the survey sought to answer included: 1. what are the perceived types of wooden food contact materials available on the Ghanaian market? 2. What are consumers’ (end-users’), sellers’, suppliers’ and manufacturers’ perceptions of wood type suitability for each available food contact item? 3. To what extent do chemical benefits and/or chemical hazards of species phytoconstituents determine indigenous wood type suitability for food contact use? Answers to these questions will clarify the underlining reasons that dictate the choice of specific wood type for the manufacture, sale and utilization of specific food contact material.

This study documents the type of wooden food contact materials available in Ghana, assesses consumers’ (end-users’), sellers’, suppliers’ and manufacturers’ perceptions of wood type suitability for each specific food contact material, enhances understanding of the factors predictive of wood type use for food contact materials, investigates the extent to which consumers (end-users), sellers, suppliers and manufacturers are aware of the chemical benefits and chemical hazards associated with the use of these wooden cookware and contributes some practical suggestions to guide national policy development on improvement in quality of available wooden food contact materials in Ghana.

## Materials and methods

### Study area

**Figure 1:**
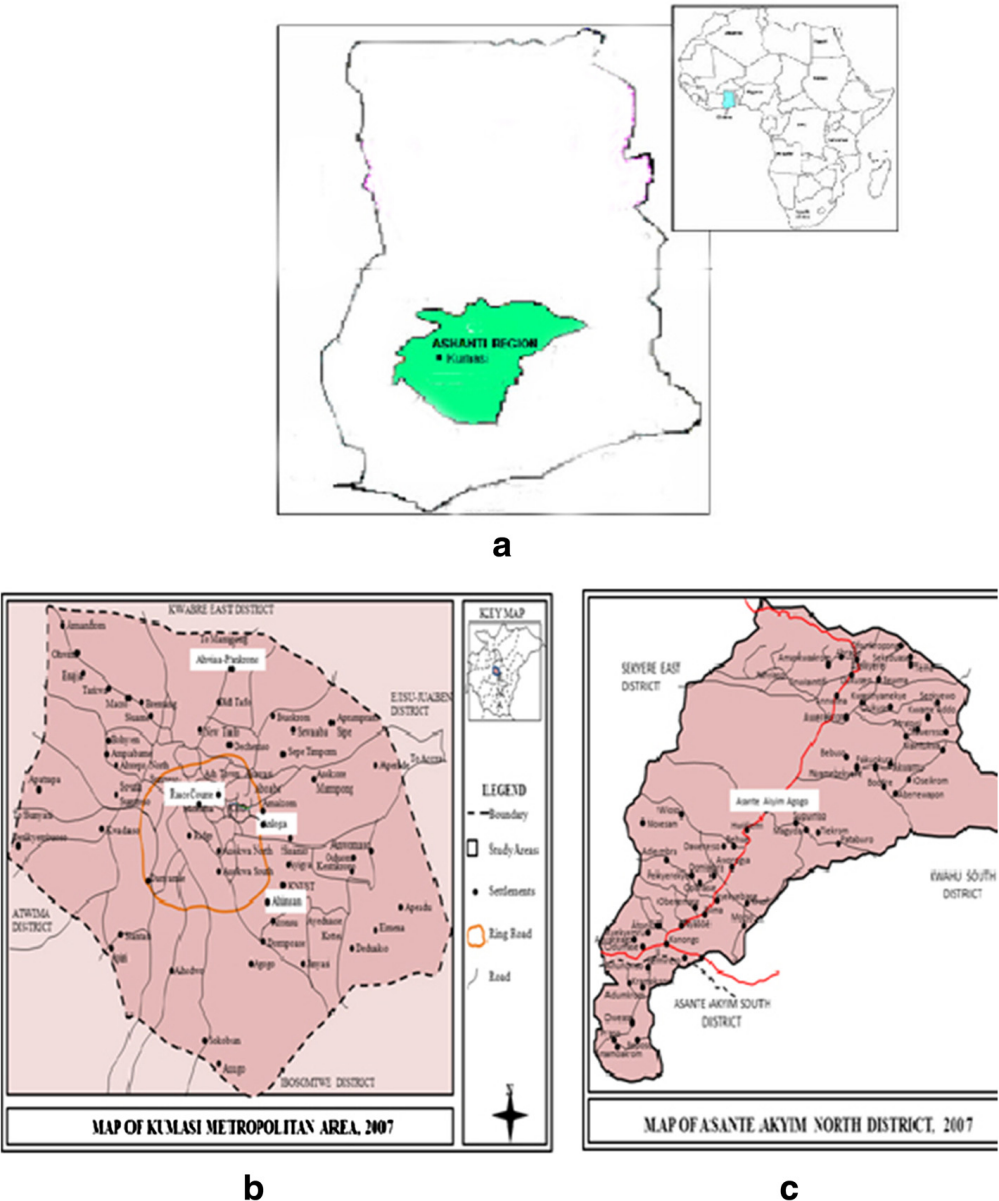
**Maps of the study area depicting a. the location of Ghana (shaded blue) in Africa and the general study area (shaded green) in the Ashanti region of Ghana; b. the exact geographical positions of surveyed suburbs within the Kumasi metropolis and c. the surveyed areas within the Asante Akyim north district.** Ashanti can also be spelt as Asante.

The study was carried out in the Kumasi Metropolitan Area and Ashanti Akyem north District both of the Ashanti Region in Ghana, from January to May 2007. The study focused on four suburbs in Kumasi Metropolitan Area (Anloga junction, Ahinsan Bus Stop, Ahwia-Pankrono and Race Course) and one surburb in the Ashanti Akyim North District where most of the wooden cookware are manufactured or sold. A map of the general study area is provided by Figure 1a. The exact geographical positions of surveyed suburbs are shown in Figure 1.

### Sampling and data collection

Cross sectional approach was adopted in conducting this research and the case study design was used because a contemporary phenomenon within real life context was being investigated. Data for the study were collected from both primary and secondary sources. Secondary data were obtained from: journals, World Wide Web, textbooks and other publications on the subject matter. Primary data were obtained from suppliers, manufacturers, retailers and consumers (end users) of different wooden cookware (mortar, pestle, grinding bowl, grinding pestle, roller, chopping board, banku ladle and wooden spoon) using closed ended questionnaires and observations.

A combination of purposive and stratified sampling techniques was used for this work. Purposive sampling technique was used to select five suburbs for the study because these are the areas well noted for the sale of the wooden cook ware. The survey respondents were stratified into manufacturers, suppliers, retailers and consumers to gain an in-depth understanding of the research topic. Suppliers purchase and sell manufactured items directly to retailers for a profit. Suppliers, in this context, do not usually transact direct business with customers as retailers do.

Since a list of the respondents (sample frame) from which a sample can be drawn, was not available a convenient sample size of 200 was selected: 25 suppliers, 25 manufacturers, 25 retailers and 125 consumers. Snow balling technique (identification through referrals from earlier subjects) was used to locate the manufacture of the wooden cookware. The consumers were located at the point of sale. After pre-testing of the questionnaires, a total of 200 respondents were administered with closed ended questionnaire.

The questionnaires were prepared in English, but local language, Twi was used to translate and communicate the content of the questionnaire where necessary since majority (about 80 percent) of the respondents were more comfortable with the local language. For interviews involving participants who could not read, the researcher asked questions with assistance from a translator and completed the survey questionnaire on their behalf with their proffered answers. Participants who needed no assistance completed the survey questionnaire themselves. The sex of participants was not noted. Each participant was asked by the questionnaire to provide personal information including age and area of residence. Participants were also asked to indicate whether or not they can identify wood species by visual inspection. Other survey questions included food contact items purchased, preferred wood species for purchased food contact item and the reason for choice of the species.

There was a non-response rate of 11.5% each from the suppliers and manufacturers even though it was explained to them that there was no legal implication of the research. It was observed that the suppliers and manufacturers were afraid to disclose their sources of wood probably because of illegal felling of trees. Fifteen percent of the consumers did not have knowledge of the type of wood species used for the various cookwares and therefore were not included in most of the analysis.

This study did not directly identify wood species. Preferred species provided by consumers (end-users), retailers, suppliers and manufacturers were reported verbatim without additional scientific identification or verification by researchers. While this approach is clearly a limitation, the study nonetheless provides a window into the diverse considerations that concerned parties make prior to wooden food contact material purchase and use. Wood type identification using current scientific technology is one of the key recommendations of this study.

### Data and statistical analysis

The nature of the research necessitated a combination of both qualitative and quantitative techniques to analyze the data. The data were analyzed using the Statistical Package for Social Science (SPSS) software (SPSS-PC for windows, version 11.0) and data were presented using cross tabulation, charts and graphs.

## Results

### Types of wooden food contact materials in the market

**Figure 2:**
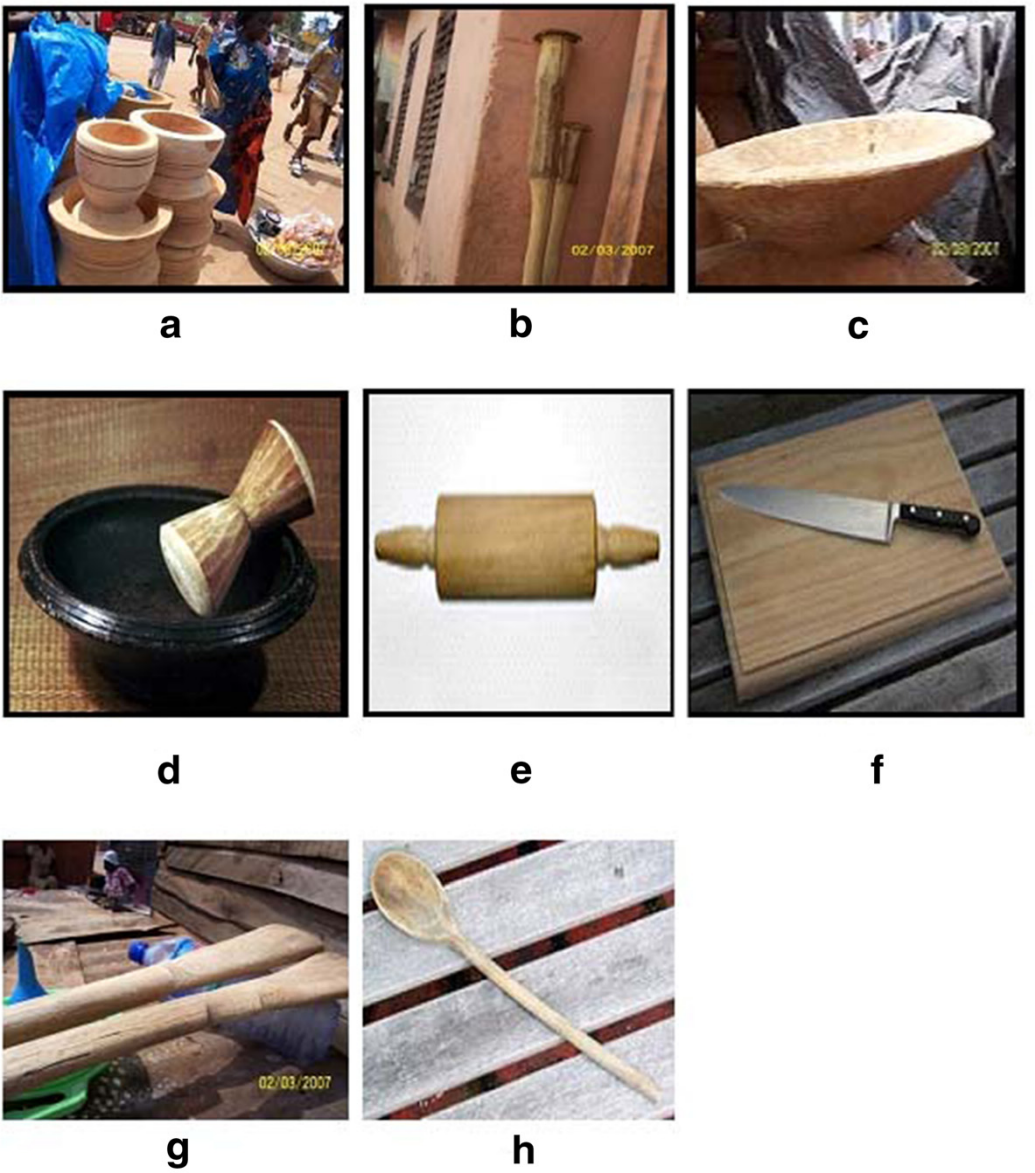
The eight wooden food contact items surveyed in the market include: a. mortar; b. pestle; c. grinding bowl; d. grinding pestle; e. roller; f. cutting/chopping board; g. banku ladle and h. wooden spoon.

The eight different wooden food contact materials identified in the market during the survey were mortar, pestle, grinding bowl, grinding pestle, roller, chopping board, banku ladle and wooden spoon (Figure 2). As observed in Figure 2, each item is unique as demonstrated by the variability in shapes and in sizes.

### Types of wood species used for the manufacture of surveyed food contact materials

As shown in Table 1, 34 species were identified to form the pool of indigenous wood commonly used for food contact purposes. The 34 species was established, based on participant responses to survey questions, as wood types used for food contact purposes in Ghana. A locally published literature has compiled, from widely scattered sources, most of the available information on indigenous species characteristics including strength, durability, shrinkage, relative availability and local names and scientific names. All information on key species characteristics listed in Table 1 was culled from this reference manual [31] with additional input from two other references [1] and [32]. All three published reports contain both local and scientific names of nearly all indigenous species.

**Table 1:** Name, key physical characteristics including strength, durability and shrinkage and relative availability of indigenous wood species commonly used for food contact cookware

**No.**	**Common name**	**Botanical name**	**Strength**	**Natural durability**	**Shrinkage**	**Forest availability**
1	Anwimfoosamina	Albizia ferruginea	Low	Low	Large	Abundant
2	Asanfran	Amphimas ferrugineus	Low/Medium	Low	Large	Average
3	Aprono	Mansonia altissima	medium	Moderate/high	Medium	Average
4	Apru	Nesorgordonia papaverifera	Medium	High	Medium	Abundant
5	Bamboo	Oxytenanthera abyssinica	Medium	Low	Small	Abundant
6	Bese	Cola nitida	Medium	Low	Medium	Below average
7	Besedua	Cola acuminata	Medium	Low	Medium	Below average
8	Danta	Cistanthera papaverifera	Low	Low	Small	Abundant
9	Emeri	Terminalia ivorensis	Medium	High	Medium	Average
10	Esa	Celtis africana	Medium	Low	Medium	Abundant
11	Esia	Combretodendron africanum	Medium	Low	Medium	Abundant
12	Essia	Petersianthus macrocarpus	Medium/high	Moderate	Large	Plentiful
13	Funtum	Funtumia elastica	Low	Low	Medium	Average
14	Gaurea	Gaurea reichard	Medium	Medium	Small	Below average
15	Hyedua	Guibourtia ehie	High	High	Medium/Large	Below average
16	Kusea	Nauclea diderrichii	Medium	Medium	Medium	Average
17	Kyere	Pterygota macrocarpa	Low/medium	Low	High	Plentiful
18	Mahogany	Khaya senegalensis	Low/medium	Moderate	Medium	Good
19	Mansonia (Aprono)	Mansonia altissima	Medium	Medium/High	Medium	Good
20	Nkutodua	Butyrospermum paradoxum	Medium	Medium	Medium	Scarce/Protected species
21	Nyamedua	Alstonia boonei	Low	Low	Medium	Plentiful
22	Odandam	-	Medium	Medium	Medium	Average
23	Odum	Chloropora excelsa	Medium	High/very high	Small/Medium	Average
24	Oframum	-	Medium	Medium	Medium	Average
25	Osina	-	Medium	Medium	Medium	Average
26	Albizia	Albizia adianthifolia	Medium	Very high	Small	Average
27	Papao	Afzelia africana	High	Very high	Small/medium	Average
28	Redwood	Sequoia sempervirens	Medium	Medium	Medium	Below average
29	Russia	Nauclea diderrichii	Medium/high	Very high	Medium/Variable	Average
30	Sapelle (Penkwa)	Entandrophragma cylindricum	Medium	Moderate	Medium	Average
31	Sese	Funtumia africana	Medium	Low	Large	Average
32	Teak	Tectoria grandis	Medium	High	Small	Plantation species
33	Wawa (Obeche)	Triplochiton scleroxylon	Low	Low	Small	Abundant
34	Wonton	Morus mesozygia	High/Very high	Low/Medium	Medium	Below average

Source: references [1,32] and [33].

### Knowledge base for indigenous wood species identification

Species identification is the necessary first step towards the potential use of a specific wood for food contact purposes. But species identification hinges primarily on the use of the physical senses to distinguish between species using prior knowledge of unique species features including distinctive colour, texture and smell of the wood. Therefore, manufacturers, suppliers, retailers and consumer perception of self-acquired knowledge essential for the identification of indigenous wood species were assessed. As shown, in Table 2, all wood suppliers, manufacturers and retailers perceive themselves as ably informed about indigenous wood species and as capable of identifying available indigenous wood species via the use of the physical senses. By contrast, 85% consumers (end-users) exhibited such capability to identify wood species (Table 2). This observation suggests that a substantial proportion of consumers (end-users) (15%) are potentially vulnerable to misrepresentation by unscrupulous retailers.

**Table 2:** Manufacturers’, suppliers’, consumers’ and retailers’ self-reported knowledge of indigenous wood species used for food contact purposes

**Response**	**Manufacturers**	**Suppliers**	**Consumers**	**Retailer**
	**(n=22) (%)**	**(n=22)(%)**	**(n=125)(%)**	**(n=25)(%)**
Knowledgeable of Wood species	100	100	85	100
Not Knowledgeable of Wood Species	0	0	15	0
Total	100	100	100	100

The acquisition of the appropriate indigenous knowledge necessary for the identification of wood species may be time-dependent. To examine the dependence of indigenous wood knowledge acquisition on time, the age of respondents and their self-reported ability to identify wood species were correlated. As expected, consumers (end-users)’ knowledge of wood species increased with age as most respondents (31%) aged 46 and above demonstrated substantial indigenous knowledge of wood (Table 3). This observation suggests that time is a necessary ingredient for the acquisition of the necessary knowledge needed for the identification of indigenous wood species.

**Table 3:** Consumers’ age and self-reported knowledge of indigenous wood species used for food contact purposes

**Age of consumers**	**Yes**	**No**	**Total**
	**(n=106) (%)**	**(n=19) (%)**	**(n=125) (%)**
15-25	19.2	4.7	23.9
26-35	16.1	4.7	20.8
36-45	19.2	4.0	23.2
46 – above	30.5	1.6	32.1
Total	85	15	100

### Knowledge of medicinal values of specific species

To examine respondent knowledge on bioactivity of species phytoconstituents or extractives, manufacturers, suppliers, retailers and consumer were asked to state knowledge of any medicinal use of the indigenous species used for food contact purposes. As shown in Table 4, 30% respondents from the combined group of manufacturers, suppliers, retailers and consumer were unaware of any medicinal values of any of the indigenous species. However 70% affirmative respondents indicated a general knowledge of species bioactivity without stating specific curative effect of specific species or its potential for migration into food. This relatively high level of affirmative respondents possibly reflects the high level of awareness of medicinal activity of extracts from most indigenous wood generated by the high prevalence of herbal medicinal practice in Ghana.

**Table 4:** Consumers’ self-reported knowledge on medicinal values of indigenous wood species

**Consumer response**	**Yes**	**No**	**Total**
	**(n=136)(%)**	**(n=58)(%)**	**(n=194)(%)**
Percentage of respondents	70	30	100

### Basis for choice of species for food contact purposes

Distinct wood features and other general factors determine species suitability for food contact use. To examine factors that influence choice of species for the manufacture, sale and use of food contact material, survey data was examined for manufacturers’, retailers’ and consumers (end-users)’ perceptions on wood type suitability for food contact use. Manufacturers rated durability (41%) more than any of the other factors including consumers’ (end-users’) demand (32%), availability (9%), cost (9%), attractive grain pattern (5%) and ease of use (5%) (Table 5). Surprisingly, the cost of wood was not as important a factor for manufacturers as it was for consumers (end-users). Unlike manufacturers, more than half of consumers (end-users) choose wood density (60%) than any other factor including colour (20%), attractive grain pattern (10%) and cost (10%).

**Table 5:** Manufacturers’ and consumers’ criteria for choosing wood species for food contact use

**Criteria**	**Manufacturers**	**Consumers**
	**(n=22) (%)**	**(n=125) (%)**
Cost (Price)	9.09	10
Attractive Grain Pattern	4.55	10
Availability	9.09	0
Customers Demand	31.82	0
Durability	40.9	0
Ease of Use	4.55	0
Density	0	60
Colour	0	20
Health Benefit/Hazard	0	0
Total	100	100

### Specific species for specific food contact items

Choice of wood species for the manufacture of food contact items was based on the broad general assessment of wood type characteristics summarized in Table 1. But distinct wood type is desirable for the manufacture of specific food contact items. To establish the extent to which specific wood type features influences the manufacture, sale and use of specific food contact items, manufacturers’, suppliers’, retailers’ and consumers’ (end-users’) perceptions on the suitability of each of the 34 species (presented in Table 1) for the manufacture of each of the eight surveyed food contact materials were separately examined.

### Preferred wood species for mortar

A mortar is a cylindrical-shaped wood stem with a hollowed-out interior used in the preparation of fufu meal or in the dehusking of boiled palm-nut fruits (Figure 2a). Due to the tremendous repetitive stress placed on the mortar by the force of impact of a pestle during use, mortar requires extremely hard and durable wood capable of absorbing the applied force without developing cracks.

Suppliers (32%), manufacturers (27%) and retailers (36%) rated Papao (Afzelia africana), a species that possesses low shrinkage and very good durability, as the best of the 15 wood species historically used to make mortars (Table 6). Although Danta (Cistanthera papaverifera) has a lower durability than Papao (Afzelia africana), consumers (end-users) chose Danta (Cistanthera papaverifera) (13%) as the best species for mortar and, in marked contrast, rated Papao (Afzelia africana) (3%) as the eighth best species for mortar. It is unknown whether consumers’ (end-users’) preference for Danta (Cistanthera papaverifera) emerged from specific determinants of choice different from that of the suppliers, manufacturers and retailers or from the lack of indigenous knowledge of wood (Table 2) already noted or from misinformation from retailers.

**Table 6:** Suppliers’, manufacturers’, retailers’ and consumers’ preferences for specific wood species for mortar

**Wood species**		**Respondents**			
**Local name**	**Scientific name**	**Manufacturers (n=22)(%)**	**Suppliers (n=22)(%)**	Consumers* (n=106)(%)	**Retailers (n=25)(%)**
Apru	Nesogordonia papaverifera	4.5	4.5	0	9.1
Asanfran	Amphimas ferrugineus	4.5	4.5	0	0
Bese	Cola nitida	4.5	0	5.7	0
Danta	Cistanthera papaverifera	4.5	18.2	13	9.1
Essia	Petersianthus macrocarpus	4.5	0	6.6	0
Kusea	Nauclea diderrichii	4.5	9.1	4.7	3
Kyere	Pterygota macrocarpa	0	0	6.6	0
Mahogany	Khaya senegalensis	0	0	7.6	0
Mansonia	Mansonia altissima	4.5	4.5	0	0
Nkutodua	Butyrospermum paradoxum	13.7	9.1	2.8	15.2
Odandam	-	0	0	7.5	0
Odum	Chlorophora excelsa	13.7	18.2	33	27.3
Oframum	-	0	0	9.7	0
Papao	Afzelia africana	27.4	31.9	2.8	36.3
Wonton	Morus mesozygia	13.7	0	0	0
Total		100	100	100	100

### Preferred wood species for pestle

A pestle is traditionally made from a 2–3 meters long tree stem with 3–6 centimeters diameter (Figure 2b). The pestle is used together with a mortar to prepare fufu meal or to dehusk palm nut fruits. The ideal pestle should be made from wood that possesses tremendous strength, have high durability and exhibits low sensitivity to moisture. The wood must be also fungi and insect resistant.

Unanimous agreement was obtained from all sur-veyed groups that Esia (Combretodendron africanum) and Wanton (Morus mesozygia) are the two preferred species for the manufacture of pestles (Table 7). Esia (Combretodendron africanum) was chosen as the best species by suppliers (67%), manufacturers (75%) retailers (70%) and consumers (end-users) (62%) while Wanton (Morus mesozygia) was chosen as the second best species by suppliers (33%), manufacturers (25%), retailers (30%) and consumers (end-users) (12%). In choosing Esia (Combretodendron africanum) and Wanton (Morus mesozygia), respondents were probably mindful of the high to medium mechanical strength of the two species. Since Esia (Combretodendron africanum) has lower strength relative to Wanton (Morus mesozygia), its choice as the best species for pestle likely stems from its high fiber quality that prevents easy breakage of the fibrous part of the fufu pestle (the portion that comes into direct contact with fufu). Interestingly, while suppliers, manufacturers and retailers limited their choice to only Esia (Combretodendron africanum) and Wanton (Morus mesozygia), consumers (end-users) included 3 other species: Mansonia (Mansonia altissima) (6%), Odum (Chlorophora excelsa) (5%), and Teak (Tectona grandis) (10%). In fact, consumers’ (end-users’) choice exhibited the largest numerical variability between species with the choice of Esia (Combretodendron africanum) (62%) being considerably higher than that of Osina (5%). But all three additional consumer chosen species (Mansonia (Mansonia altissima), Odum (Chlorophora excelsa), and Teak (Tectona grandis)) are unified by very high natural durability and medium relative strength. Therefore, it is unknown whether these 3 additional alternative consumer preferences reflect their relatively low level of indigenous knowledge of wood species or reflect motivational factors different from that of the other groups. It is also unknown whether the inclusion of these 3 additional species by consumers (end-users) stem from experiential knowledge garnered from the actual use of pestles made from the specified species or from different preferences of end-users with different socio-economic backgrounds.

**Table 7:** Suppliers’, manufacturers’, Retailers’ and Consumers’ preferences for specific wood species for pestle

**Local name**	**Botanical name**	**Manufactures (n=22)(%)**	**Suppliers (n=22)(%)**	Consumers* (n=106)(%)	**Retailers (n=25)(%)**
Esia	Combretodendron africanum	75	67	62	70
Mansonia	Mansonia altissima	0	0	6	0
Odum	Chlorophora excelsa	0	0	5	0
Osina	-	0	0	5	0
Teak	Tectona grandis	0	0	10	0
Wanton	Morus mesozygia	25	33	12	30
Total		100	100	100	100

*Excludes 19 respondents who claimed not to be knowledgeable in wood species.

### Preferred wood species for grinding bowls

Grinding bowls are carved cylindrical bowl-shape piece of wood used primarily to blend pepper, onion and tomato and to prepare mashed plantain, yam, or cocoyam meal known traditionally as eto (Figure 2c). Other local uses for the grinding bowl including eating, drawing water and storing fruits and vegetables are also known. Although the wooden grinding bowls have been largely replaced by the cheaper earthenware alternative, a market still exists for this cooking utensil. However, no manufacturer of grinding bowl could be identified for this survey.

Suppliers differed from retailers and consumers (end-users) by choosing Mahogany (Khaya senegalensis) (47%) as the best species (Table 8). The choice of Mahogany (Khaya senegalensis) for the grinding bowl is likely attributable to the species’ high mechanical strength and its beneficial bioactivity. In contrast, consumers (end-users) chose both Wawa (Triplochiton scleroxylon) (35%) and Nyamedua (Alstonia boonei) (35%) while retailers agreed with consumers’ (end-users’) choice of Nyamedua (Alstonia boonei) (45%) as the best species. Both Wawa (Triplochiton scleroxylon) and Nyamedua (Alstonia boonei) possess low intrinsic strength and low natural durability and it is difficult to rationalize the choice of both species by consumers (end-users) for a food contact item that requires high abrasion resistance and tremendous mechanical strength. However, all three groups (suppliers (27%), consumers (end-users) (20%), and retailers (28%)) agreed that Odum (Chlorophora excelsa), a species with very high durability, was the second best species for the manufacture of grinding bowls.

**Table 8:** Suppliers,’ retailers’ and consumers’ preferences for specific wood species for grinding bowl

**Local name**	**Botanical name**	**Suppliers (n=22)(%)**	Consumers* (n=106)(%)	**Retailers (n=25)(%)**
Kyere	Pterygota macrocarpa	0	4	7
Mahogany	Khaya senegalensis	47	6	13
Nyamedua	Alstonia boonei	0	35	45
Odum	Chlorophora excelsa	27	20	28
Teak	Tectona grandis	0	0	7
Wawa	Triplochiton scleroxylon	13	35	0
Wonton	Morus mesozygia	13	0	0
Total		100	100	100

*Excludes 19 respondents who claimed not to be knowledgeable in wood species.

### Preferred wood species for grinding pestle

A grinding pestle is a short dumbbell-shaped item traditionally used together with a grinding bowl to blend pepper, tomatoes and onions sauce through the simple rhythmic movement of the wrist (Figure 2d). Due to the high level of attrition and wear originating from the repetitive frictional contact with the grinding bowl, high abrasion resistance wood is desirable. No manufacturer of the grinding pestle could be located for this survey. While majority of suppliers (46%) preferred Mahogany (Khaya senegalensis), consumers (end-users) (37%) and retailers (44%) settled on Nyamedua (Alstonia boonei) as the best species for the grinding pestle (Table 9). While Nyamedua’s (Alstonia boonei) relative availability remains high, consumer preference for its use for the grinding pestle cannot, however, be readily accounted for by its relatively low intrinsic strength and comparatively low natural durability.

**Table 9:** Suppliers’, manufacturers’, retailers’ and consumers’ preferences for specific wood species for grinding pestle

**Wood species**		**Respondents**		
**Local name**	**Scientific name**	**Suppliers (n=22)(%)**	Consumers* (n=106)(%)	**Retailers n=25)(%)**
Kyere	Pterygota macrocarpa	0	4.8	8
Mahogany	Khaya senegalensis	46	8.8	12
Nyamedua	Alstonia boonei	0	33.6	44
Odum	Chlorophora excelsa	27.2	19.2	28
Teak	Tectona grandis	0	0	8
Wawa	Triplochiton scleroxylon	13.4	33.6	0
Wonton	Morus mesozygia	13.4	0	0
Total		100	100	100

*Excludes 19 respondents who claimed not to be knowledgeable in wood species.

### Preferred wood species for roller

Rollers are cylindrical shaped wood with handles at each end used primarily to blend and flatten flour dough (Figure 2e). Ideally, rollers require durable wood with high density. Redwood (Sequoia sempervirens), a high den-sity wood of medium durability, was the overwhelming favourite of retailers (68%) compared to the 3 other preferred species (Table 10). Retailers also considered Apru (Nesogordonia papaverifera) (24%), Danta (Cistanthera papaverifera) (4%) and Teak (Tectona grandis) (4%). Most consumers (end-users) (32%) preferred Redwood (Sequoia sempervirens) more than the 4 additional species including Odum (Chlorophora excelsa) (29%), Mahogany (Khaya senegalensis) (19%), Danta (Cistanthera papaverifera) (13%) and Teak (Tectona grandis) (7%). Although manufacturers considered 8 species suitable for rollers, none of them included Redwood (Sequoia sempervirens). Manufacturers preferred Mahogany (Khaya senegalensis) (22%) and Danta (Cistanthera papaverifera) (18%) as the 2 best species. Just like manufacturers, suppliers also chose Mahogany (Khaya senegalensis) (32%) and Danta (Cistanthera papaverifera) (46%) as the 2 best species.

**Table 10:** Suppliers’, manufacturers’, retailers’ and consumers’ preferences for specific wood species for roller

	**Wood species**	**Respondents**			
**Local name**	**Scientific name**	**Manufacturers (n=22)(%)**	**Suppliers (n=22)(%)**	Consumers* (n=106)(%)	**Retailers (n=25)(%)**
Albizia	Albizia adianthifolia	4.5	0	0	0
Apru	Nesogordonia papaverifera	0	0	0	24
Danta	Cistanthera papaverifera	18.3	45.5	13	4
Hyedua	Guibourtia ehie	4.5	0	0	0
Kyere	Pterygota macrocarpa	13.8	0	0	0
Mahogany	Khaya senegalensis	22.2	31.8	19	0
Odum	Chlorophora excelsa	0	0	28.5	0
Papao	Afzelia africana	13.8	0	0	0
Redwood	Sequoia sempervirens	0	0	32.1	68
Sapelle	Entandrophragma cylindricum	9.1	0	0	0
Teak	Tectona grandis	13.8	22.7	7.4	4
Total		100	100	100	100

*Excludes 19 respondents who claimed not to be knowledgeable in wood species.

### Preferred wood species for cutting/chopping board

The cutting or chopping boards constitute one of the most ubiquitous wooden cookware in the kitchen made from any reasonably sized piece of wood (Figure 2f). Apparently all the groups preferred different species. Retailers (48%) chose Redwood as the best of 4 species (Table 11). Manufacturers chose Danta (Cistanthera papaverifera) (27%) as the most preferred of 7 species. Suppliers chose Mahogany (Khaya senegalensis) (40%) over 3 other species while consumers (end-users) chose Odum (Chlorophora excelsa) (22%) over 6 other species. It is unknown whether such broad spectrum of choice from all surveyed groups reflects individual and group differences in the constitution of a good cutting/chopping board or reflects a general laxity in the use of any available wood surface as cutting/chopping board.

**Table 11:** Suppliers’, manufacturers’, retailers’ and consumers’ preferences for specific wood species for cutting/chopping board

	**Wood species**	**Respondents**			
**Local name**	**Scientific name**	**Manufacturers (n=22)(%)**	**Suppliers (n=22)(%)**	Consumers* (n=106)(%)	**Retailers (n=25)(%)**
Albizia	Albizia adianthifolia	18.2	20	0	0
Danta	Cistanthera papaverifera	27.2	0	0	20
Hyedua	Guibourtia ehie	0	0	17	0
Kyere	Pterygota macrocarpa	13.7	0	0	0
Mahogany	Khaya senegalensis	4.5	40	15	8
Mansonia	Mansonia altissima	0	30	0	0
Odandam	-	0	0	6.6	0
Odum	Chlorophora excelsa	0	0	21.7	0
Oframum	-	0	0	8.5	0
Papao	Afzelia africana	18.2	0	0	8
Redwood	Sequoia sempervirens	0	0	16.8	48
Sapelle	Entandrophragma cylindricum	13.7	0	0	0
Teak	Tectona grandis	4.5	10	0	16
Wawa	Triplochiton scleroxylon	0	0	14.4	0
Total		100	100	100	100

* Excludes 19 respondents who claimed not to be knowledgeable in wood species.

### Additional selection criteria for banku ladles and wooden spoons

**Figure 3:**
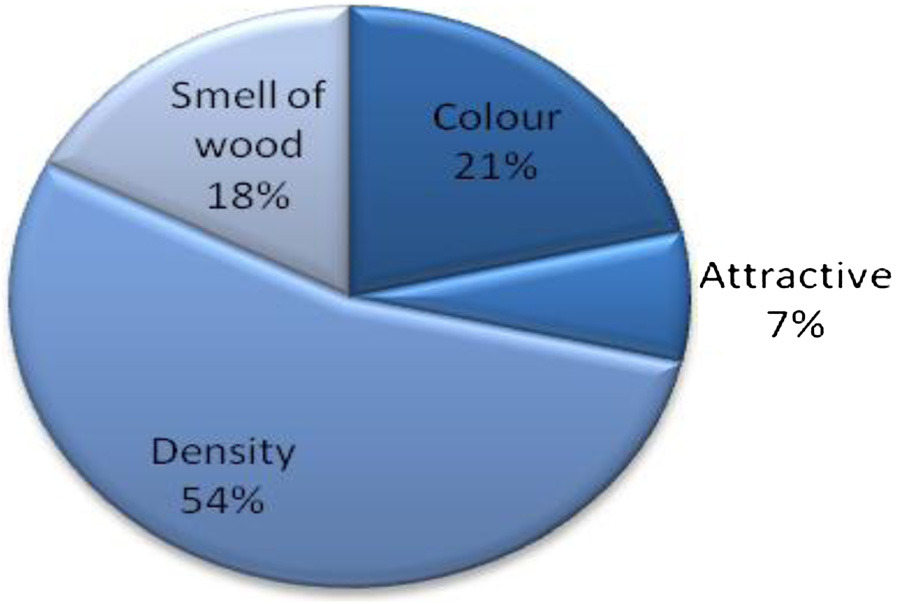
Manufacturers’ and consumers’ (end-users’) additional criteria for choosing wood species for banku ladles and wooden spoons.

Banku ladles are large spoon-shaped wood used primarily to stir and knead cornmeal during the preparation of the local banku meal (Figure 2g). Although the wood selection criteria enumerated earlier (Table 5) are generally applicable to the available broad group of wooden food contact items, banku ladles and wooden spoons were found to require additional distinct specifications. As shown in Figure 3, consumers (end-users) preferred wood that has low density (54%); is non-coloured (21%) (by implication woods that will not stain food with coloured phytochemicals); has attractive grain pattern (7%) and possesses an attractive smell (18%).

### Preferred wood species for banku ladles

To permit efficient turning of the banku ladle and effective kneading of the cornmeal during food preparations, lighter but firmer wood is preferred. Unanimous agreement was obtained from suppliers (50%), retailers (50%) and consumers (end-users) (43%) that Wawa (Triplochiton scleroxylon) best satisfies the additional criteria enumerated in Figure 3 and that Wawa is the most desirable species for Banku ladles (Table 12). Wawa is a light wood with low strength and low durability and its preference for Banku ladles probably stems from its lightness.

**Table 12:** Suppliers’, manufacturers’, retailers’ and consumers’ preferences for specific wood species for banku ladles

**Wood species**		**Respondents**		
**Local name**	**Scientific name**	**Suppliers (n=22)(%)**	Consumers* (n=106)(%)	**Retailers (n=25)(%)**
Nyamedua	Alstoniaboonei	0	11.2	20
Besedua	Cola acuminate	0	5.6	0
Odum	Chlorophora excels	0	5.6	10
Wawa	Triplochiton scleroxylon	50	43.2	50
Kyere	Pterygota macrocarpa	0	11.2	0
Sese	Funtumia africana	0	8.8	0
Bamboo	Oxytenanthera abyssinica	18.2	8.8	0
Funtum	Funtumia elastica	0	5.6	0
Danta	Cistanthera papaverifera	22.7	0	20
Anwimfoosamina	Albizia ferruginea	9.1	0	0
Total		100	100	100

*Excludes 19 respondent who claimed not to be knowledgeable in wood species.

### Preferred wood species for wooden spoons

The relatively small sizes of the ubiquitous wooden spoons suggest that they could, in principle, be crafted from any reasonably-sized piece of wood (Figure 2h). And this rationale likely accounts for the choice of multiple preferred species by all respondent groups (Table 13). Danta (Cistanthera papaverifera) was best of the 11 species manufacturers (23%) chose as suitable for spoons. Manufacturer’s choice of Danta (Cistanthera papaverifera) was echoed by majority of suppliers (37%) as the best choice of 5 preferred species. Most retailers (32%) favoured Odum (Chlorophora excelsa) out of the 6 preferred species that also included Danta (Cistanthera papaverifera) (12%). Unsurprisingly, consumers (end-users) did not consider Danta (Cistanthera papaverifera), but chose Wawa (Triplochiton scleroxylon) (43%) out of 8 preferred species. Consumers’ (end-users’) choice for Wawa (Triplochiton scleroxylon) was perhaps motivated by the lightness of the species. In an observation supporting earlier assertion of different consumer preferences from that of manufacturers’, suppliers’ and retailers, two of consumers (end-users) choices Besedua (Cola acuminata) and Funtum (Funtumia elastica) were species the 3 other groups have jointly considered unsuitable for wooden spoons.

**Table 13:** Suppliers’, manufacturers’, retailers’ and consumers’ preferences for specific wood species for wooden spoon

**Wood species**		**Respondents**			
**Local name**	**Scientific name**	**Manufacturers (n=22)(%)**	**Suppliers (n=22)(%)**	Consumers* (n=106)(%)	**Retailers (n=25)(%)**
Albizia	Albizia adianthifolia	4.5	22.3	0	0
Apru	Nesogordonia papaverifera	0	0	0	4
Bamboo	Oxytenanthera abyssinica	4.5	0	8.8	0
Besedua	Cola acuminate	0	0	5.6	0
Danta	Cistanthera papaverifera	22.9	37.2	0	12
Funtum	Funtumia elastica	0	0	5.6	0
Guarea	Guarea reichard	9.1	0	0	0
Kyere	Pterygota macrocarpa	9.1	0	11.2	0
Mahogany	Khaya senegalensis	9.1	22.3	0	12
Nyamedua	Alstonia boonei	0	0	11.2	0
Odum	Chlorophora excelsa	9.1	0	5.6	32
Papao	Afzelia africana	18.2	0	0	24
Redwood	Sequoia sempervirens	0	0	0	4
Sese	Funtumia africana	4.5	0	8.8	0
Teak	Tectona grandis	4.5	9.1	0	12
Wawa	Triplochiton scleroxylon	4.5	9.1	43.2	0
Total		100	100	100	100

## Discussion

In this study, a comprehensive assessment of the factors that determine the suitability of specific indigenous wood species for specific food contact materials was conducted using a survey research on a representative community sample of manufacturers, suppliers, retailers and consumers (end-users) of wooden food contact materials. This approach was reasoned to comprehensively elucidate the underlining reasons that dictate the choice of specific wood type for the manufacture, sale and utilization of specific food contact material.

The critical first step towards the potential use of a specific wood for food contact purposes is species identification. Wood species identification is important because: the intrinsic suitability of any wood for food contact purposes is species-dependent; the dose and types of beneficial or deleterious wood phytoconstituents that may augment or preclude its food contact use are species-specific; the commercial values of wooden food contact materials are species-dependent. The reason for which you need reliable identifications in order to make conclusions.

The reliance of species identification on acquired indigenous wood knowledge encompassing specific colour, distinctive texture, unique grain pattern and characteristic smell of species suggest guess-work is involved in the distinction between closely related species or in the identification of different species that have similar physical features [33]. In addition to the demonstrated low consumer indigenous knowledge of wood and its reflection in the disparate choices of species for selected food contact items made by consumers’ (end-users’) relative to that made by the combined group of manufacturers, suppliers and retailers, it is reasonable to conclude that visual identification of species cannot be reliably used by end-users for species distinction of marketed products. Although older consumers (end-users) will have much more experience and a relatively higher aptitude to make reliable purchases of historically proven species, most consumers (end-users) will be susceptible to species misrepresentation by unscrupulous retailers. Since the reliability of wood species identification through indigenous knowledge ultimately impinges on food quality and food safety, the implication of any deficiency in wood species identification, as observed in 15% of consumers (end-users), is not just the loss of chemical benefits from beneficial species but also potential exposures to chemical hazards from potentially toxic species phytoconstituents from toxic historically unproven species [22-28]. For example, ethanolic extract of the stem-bark of Nyamedua (Alstonia boonei) impairs reproduction in male albino rats [34] and contributes to nephrotoxicity in guinea pigs [35]. But the continued use of Nyamedua (Alstonia boonei) for food contact purposes belies its potential for toxicity if indeed its wood has similar phytoconstituent composition as its stem-bark.

For all types of food contact materials, species availability was found to be a key factor that determines the range of marketable products. But species availability has been adversely affected by rapid deforestation and the scarcity of some species including Odum (Chlorophora excelsa), Mahogany (Khaya senegalensis), and Sapele (Entandrophragma cylindricum) has placed a price premium on them and has also necessitated the use of alternatives species including Teak (Tectona grandis) and Kyere (Pterygota macrocarpa) [36]. But it is unknown to end-users, through the scant available historical knowledge, whether or not the lesser-known or lesser utilized alternative wood sources or the newly introduced species in response to the dwindled pool of previously available ones represent a more or a less physiologically beneficial alternative. These critical questions can convincingly be settled by scientific research that thoroughly examines the health-related consequences of wood-species derived bioactive compounds. Nevertheless, for all wood species in use for food contact purposes, an important context-related question is: what are the criteria for wood type suitability for food contact use? Despite the heterogeneity in shapes and sizes of wooden food contact materials, some common suitability criteria proffered as answers to this question by manufacturers and consumers (end-users) include durability, consumers’ (end-users’) demand, availability, price, attractive grain pattern, odour, wood density, colour and ease of use. It is also apparent that consumers (end-users) preferred aesthetically pleasing wood at relatively low cost, as demonstrated by their choice of colour and attractive grain pattern.

Although the application of these suitability criteria, on a historical basis, by manufacturers and consumers (end-users) has successfully established distinct species of wood in current use for food contact purposes, a common observation is that none of the enumerated suitability factors included potential chemical benefits or likely chemical hazards of wood phytoconstituents. This observation emphasizes the notion that the choice of wood for the manufacture of food contact materials in Ghana is dictated primarily by reasons other than the chemical benefits or the chemical hazards presented by wood phytoconstituents. Despite increasing recognition of the possible chemical transfers from wood to food [12-17], respondent groups failed to indicate any awareness of chemical benefits or chemical hazards associated with the use of wood food contact materials. In fact given the opportunity to consider this health-related option in the questionnaire, neither chemical benefits with implied knowledge of medicinal bioactivities of wood phytoconstituents nor chemical hazards with implied toxicological knowledge of wood chemicals were chosen by respondents in their respective wood type suitability assessment. In all cases, respondents made erroneous assumptions that all species of wood used for the manufacture of food contact materials in the market were non-toxic and that no direct negative health-related consequences could result from their use. The study, therefore, demonstrates in a community setting, that the knowledge of manufacturers, suppliers, retailers and consumers (end-users) on benefits and hazards of wood phytoconstituents or extractives of food contact items is abysmally low. And this observation translates into species suitability determinant system that gives little or no credence to the impact of potentially beneficial or toxic phytoconstituents migrant from wood to food on human health. Taken together, these observations support the prevailing assumption that does not consider domestic wooden food contact items as viable avenues for the transfer of beneficial and/or toxic active principles to food.

The observation that Danta (Cistanthera papaverifera) and Mahogany (Khaya senegalensis) are the only two species used for the manufacture of all surveyed food contact items is particularly interesting because neither the strength nor the durability of both species are exceptionally high to justify the observed multiple usage. It is reasonable to assume that the phytoconstituents of Mahogany (Khaya senegalensis), valued for its myriad curative properties in ethnomedicinal practices, likely accounts for its preferences for all food contact items. In fact, Mahogany (Khaya senegalensis)-derived bioactive extracts are produced and marketed nation-wide as dietary supplements [37].

The stem-bark extracts of Mahogany (Khaya senegalensis) contain alkaloids, saponins, tannins, flavonoids [38] and limonoids of angolensates, ring D-opened limonoids and mexicanolides. [39,40]. Some of these phytoconstiuents are also present in the wood of Mahogany (Khaya senegalensis) at relatively lower concentrations. The possible phytochemical migration from Mahogany (Khaya senegalensis) wood to food is unknown. Therefore, assertive conclusions cannot be made on whether or not Mahogany (Khaya senegalensis) phytoconstituents leach into food. The recommended detailed scientific investigation into phytochemical migrants to food is long overdue.

But given the general lack of awareness of respondent groups to potential chemical benefits of species, it is also doubtful that the preferences for Mahogany (Khaya senegalensis) for multiple items are informed by its numerous medicinal activities. This notion is perhaps supported by the additional choice of Danta (Cistanthera papaverifera), a species with no known or widespread medicinal value, for multiple uses. On an item-by-item basis, banku ladle and wooden spoons have a shared utility, namely, their use for prolonged periods at high temperatures. As a consequence, both items can potentially transfer high quantitative levels of bioactive phytochemicals to food at the earlier times of use (mostly between the first and the third time of use). Banku ladles are used mostly in aqueous environments and at temperatures high enough to facilitate the transfer of polar phytochemical functionalities to food [41]. Similarly, wooden spoons used at high temperatures in both aqueous and nonpolar milleu facilitate the transfer of a large spectrum of phytochemical functionalities to food [41].

It is also interesting to note that grinding pestles undergo significant attrition and wear during use and may likely leave residual ground wood in the food during contact. Wood phytoconstitutuent migrants resulting from the grinding action of the grinding pestle are probably responsible for the impartation of the unique taste to ground pepper and tomato sauce prepared from the combined use of grinding pestle and grinding bowl. Published reports on the hygienic suitability of wood as cutting/chopping board are contradictory with some studies suggesting comparatively higher bacteria accumulation and retention even after cleaning [42-44] and others positing that the combination of hygroscopic properties and antimicrobial bioactivities of wood extractives enable satisfactory hygienic performance [45,46]. It is apparent from both sides of the argument that the cutting/chopping board will exhibit the highest tendency, among the eight studied food contact items, to act as an incubator for bacteria growth and will probably facilitates the transfer of more pathogenic bacteria to food than any other wooden food contact item [3,4,9,45,47-52]. The use of Mahogany (Khaya senegalensis) for a chopping board can likely be accounted for by its myriad biological activities including potential antimicrobial activities. Antimicrobial activities have not been reported in the wood of Mahogany (Khaya senegalensis). But since the wood contains secondary metabolites such as alkaloids, saponins, tannins and flavonoids that are frequent hallmarks of antibacterial activities, it can be reasonably assumed that Mahogany (Khaya senegalensis) wooden food contact items may possess antimicrobial activities. It is unknown whether the phytoconstituents of Danta (Cistanthera papaverifera) and Odum (Chlorophora excelsa), the two most preferred species for chopping boards, have intrinsic antibacterial properties. These observations emphasize the continuing importance of the use of chemically safe wood for food contact purposes.

Although respondent groups failed to indicate, overtly, any knowledge of possible wood phytoconstitutuent migration to food, the general practice of “priming” new food contact materials seems to contradict their stated perceived lack of knowledge in this area. New food contact items are “primed” prior to use by immersion in hot water and/or thorough washing with hot water followed by a purposeful disposal of the bits of food that makes first contact with the wood. People “prime” their utensils to reduce the amount of chemical compounds that transfers to foods at the initial stages of use. “Priming” is by default a practical demonstration of possible phytoconstituent migration to food. Implicit in this general practice is the recognition that some potentially toxic or unpleasant chemical substances might transfer from wood to the food at higher doses during the early stages of use of the food contact item. And that the practice of “priming” reduces the concentrations of such chemical substances to low, possibly non-toxic, levels prior to regular use. This observation is demonstrated by the reduction in the initial “bitter” taste that wood phytoconstituents impart to the food that makes direct contact with the wooden cookware. Despite its high prevalence in Ghana, the practice, however, lacks direct report or reference in the literature.

The transfers of chemicals from wood to food represents an area in which published research information is meager and thus, the nature and pattern of such chemical migration remain largely undescribed. However, mechanistic sketches of phytochemical migration from wood to food [15,53] can be deduced from that of the most thoroughly studied example of chemical transfers to food, namely that of plastic food packages [54-57]. Net chemical intake by humans from food that has made prior contact with wood is likely dependent on the species-specific concentration and on the rate of the chemical compound’s diffusional transfer to food [58]. Wood phytoconstituent migrants may elicit a wide range of beneficial and/or deleterious physiological responses in humans even at very low doses [18,59-63]. So that low beneficial or toxic phytoconstituent concentrations in the wood species coupled with the phytochemicals low diffusional transfer rates to food may be just sufficient, in some cases, to attain biological significant concentrations of some chemical substances in the human body [53]. These observations underline the importance of using toxicologically safe and/or chemically beneficial species for food contact purposes. A critical unanswered question that remains within this context is whether or not the net chemical intake by humans from wooden food contact items constitutes a problem that warrants the level of caution alluded to in this study. This field of study is unquestionably a fertile one for research and definitive resolution of this and many related seminal topics and questions require carefully controlled experimentations.

For a start, food contact item regulatory bodies and researchers in Ghana can initiate biochemical research that uses molecular features including cellular wood anatomy to accurately identify all species in current use for food contact purposes [33,64-66]. This approach will eliminate much of the guess-work and subjectivity associated with species identification by direct visual inspection and will pave the way for the complete chemical and biological characterization of all indigenous species in current use for food contact purposes. The documentation of the chemical compositional differences, including the presence of specific bioactive extractives or group of bioactive extractive among species, will provide a better understanding of species commonalities and differences [67-69]. Chromatographic separation, via Thin Layer and Column Chromatography, followed by phytoconstituent isolation and spectroscopic-methods based structural characterization of key bioactive constituents will lead to the identification of the molecular types of all wood phytoconstituents on a species-by-species basis and a classification of these molecular groups into potentially medicinal or toxic active principles [2,70-72]. GC/MS, LC/MS and HPLC/MS analyses will furnish the relative concentration of known bioactive phytoconstituents in all available species [73]. Analytical chemistry methodologies will provide the baseline concentration of wood phytochemical migrant in common local foods prepared in the normal manner using specific wood species of food contact items as well as establish the human exposure levels to specific types of wood phytoconstituents migrants. Molecular biological studies will establish whether the species-specific dose of wood phytochemical migrants in food is sufficient to trigger any biological response and if it is, will decipher whether the physiological response is beneficial or deleterious. And if warranted, mechanistic studies will identify the molecular target(s) and the biological mechanisms underlying the putative beneficial or toxicological action of specific phytochemical migrants.

In the long term practical sense, the dependence of wood species use on oral transmission of historical knowledge is unacceptable because cultural and economic acculturation pressures from modern society may endanger this practice. Regulatory bodies in Ghana can document and preserve this traditional historical knowledge in a curated database, cataloguing each species’ distinctive features and providing a comparative perspective on differences in structural features between species, the biological and chemical differences within and between species as well as specifying the potential for chemical hazard or the likely health benefits on a species-by-species basis. The adverse effect of deforestation on species availability suggests that the use of chemically safe and beneficial alternative species from sustainably managed forest should be encouraged by regulatory bodies. To facilitate informed purchasing decision by consumers (end-users), regulatory bodies must insist that retailers label each food contact item offered for sale with both the indigenous and scientific names of species.

Coupled with the comprehensive scientific analyses already suggested, this approach will provide a scientific qualitative knowledge base that will safeguard the indigenous knowledge on old and new wood species that are chemically safe and phytochemically beneficial for food contact uses and will permit the seamless translation of indigenous knowledge consistent with scientific understanding of food safety among wooden food contact items.

## Conclusion

The continued use of specific wood species for crafting unique food contact items is dictated by historical precedents, distinct species physical and aesthetic features and by species availability. The observed lack of consumer appreciation for potential chemical benefits and/or likely chemical hazards via the possible transfer of wood phytoconstituents to food offers an interesting dynamics that need to be addressed by all concerned parties.

## Competing interests

The authors declare that they have no competing interests.

## Authors’ contributions

JKM analyzed the collated data and prepared the final manuscript. EA and DA developed and designed the study. GOA conducted field survey work and collected the data. All authors read and approved the final manuscript.

## References

[B1] Irvine FR Woody Plants of Ghana 1961 With special reference to their uses: London, Oxford University Press

[B2] Buchanan MA Browning BL The Chemistry of Wood 1963 New York

[B3] Bowyer JL Shmulsky R Haygreen JG Forest products and wood science, An introduction 2007 5 UK: Blackwell Publishing Ltd

[B4] Sjostrom E Wood Chemistry. Fundamentals and Applications 1993 Second San Diego: Academic Press

[B5] Haygreen JG Bowyer JL Forest Products and Wood Science, an Introduction 1996 Ames, IA: Iowa State University Press

[B6] Pometti CL Palanti S Pizzo B Charpentier JP Boizot N Resio C Saidman BO Durability of five native Argentine wood species of the genera Prosopis and Acacia decayed by rot fungi and its relationship with extractive content Biodegradation 2010 21 5 753 760 10.1007/s10532-010-9340-5 20195704

[B7] Rowe JW Extractives in eastern hardwoods: a review.Gen. Tech. Rep. FPL-18 1979 Madison, WI: U.S. Department of Agriculture, Forest Service, Forest Products Laboratory

[B8] Pietarinen S Willlfor S Sjopholm R Holmbom B Bioactive phenolic substances in important tree species, Part 3 Knots and stemwood of Acacia crassicarpa and A. mangium. Holzforschung 2004 59 94 101 104

[B9] Schulz H Wood in contact with food. Is wood bactericidal? Fleischwirtschaft 1995 75 7 864 868

[B10] Schultz TP Nicholas DD Naturally durable heartwood: evidence for a proposed dual defensive function of the extractives Phytochemistry 2000 54 1 47 52 10.1016/S0031-9422(99)00622-6 10846746

[B11] Doussot F De Jéso B Quideau S Pardon P Extractives content in cooperage oak wood during natural seasoning and toasting; influence of tree species, geographic location, and single-tree effects J Agric Food Chem 2002 50 21 5955 5961 10.1021/jf020494e 12358465

[B12] Bradley EL Honkalampi-Hämäläinen U Weber A Andersson MA Bertaud F Castle L Dahlman O Hakulinen P Hoornstra D Lhuguenot JC Mäki-Paakkanen J Salkinoja-Salonen M Speck DR Severin I Stammati A Turco L Zucco F Von Wright A The BIOSAFEPAPER project for in vitro toxicity assessments: preparation, detailed chemical characterization and testing of extracts from paper and board samples Food Chem Toxicol 2008 46 7 2498 2509 10.1016/j.fct.2008.04.017 18508176

[B13] Bradley EL Stammati A Salkinoja-Salonen M Andersson M Bertaud F Hoornstra D Zucco F Weber A Turco L Traussnig H Hakulinen P Speck DR Von Wright AJ Honkalampi-Hamalainen U Maki-Paakkanen J Severin I Lhuguenot JC Dahlman O Test procedures for obtaining representative extracts suitable for reliable in vitro toxicity assessment of paper and board intended for food contact Food Addit Contam Part A Chem Anal Control Expo Risk Assess 2010 27 2 262 271 10.1080/02652030903232746 20013449

[B14] Honkalampi-Hämäläinen U Bradley EL Castle L Severin I Dahbi L Dahlman O Lhuguenot JC Andersson MA Hakulinen P Hoornstra D Mäki-Paakkanen J Salkinoja-Salonen M Turco L Stammati A Zucco F Weber A von Wright A Safety evaluation of food contact paper and board using chemical tests and in vitro bioassays: role of known and unknown substances Food Addit Contam Part A Chem Anal Control Expo Risk Assess 2010 27 3 406 415 10.1080/19440040903401358 20087806

[B15] Offen C Becker P Unusual and non-traditional types of wood as food contact materials, and the implication for food safety 2002 http://www.foodbase.org.uk/admintools/…/617-1-1040_A03024_25.pdf

[B16] Kirkeskov L Witterseh T Funch LW Kristiansen E Molhave L Hansen MK Knudsen BB Health evaluation of volatile organic compound (VOC) emission from exotic wood products Indoor Air 2009 19 1 45 57 10.1111/j.1600-0668.2008.00560.x 19191927

[B17] Von Wright A Bradley E Honkalampi-Hämäläinen U Castle L Weber A Salkinoja-Salonen M Andersson M Hoornstra D Lhuguenot JC Severin I Dahbi L Stammati A Dahlman O Turco L Zucco F Safety Evaluation of Food contact paper and board using Chemical Tests and in vitro Bioassays-The role of known and unknown substances Food Addit Contam 2010 27 3 406 415 10.1080/19440040903401358 20087806

[B18] Ayars GH Altman LC Frazier CE Chi EY The toxicity of constituents of cedar and pine woods to pulmonary epithelium J Allergy Clin Immunol 1989 83 3 610 8 10.1016/0091-6749(89)90073-0 2926083

[B19] Ejechi BO Growth inhibition of Pseudomonas aeruginosa and Enterococcus faecalis by crude extractives of Mansonia altisima timber sawdust Curr Microbiol 1996 32 5 297 298 10.1007/s002849900053 8857275

[B20] Durzan DJ Arginine, scurvy and Cartier's "tree of life" J Ethnobiol Ethnomed 2009 http://www.ethnobiomed.com/content/5/1/5 10.1186/1746-4269-5-5 PMC2647905 19187550

[B21] Mollet LML Cadwallader KR Handbook of meat, poultry and seafood quality 10.1002/9780470277829.ch15/summary2007

[B22] Deroubaix G Quality and safety scheme for wood in food contact Proceedings of the 3rd International Wood Preservation Symposium The Challenge - Safety and Environment 1995 163 176

[B23] Banner R Potentially toxic woods Musical Instrument Makers Forum 1998 http://www.mimf.com

[B24] Hausen BM Woods Injurious to Human Health: A Manual 1981 Berlin: Walter de Gruyter & Co

[B25] Jagels R Health hazards of natural and introduced chemical components of boatbuilding woods Am J Ind Med 1985 8 1 241 251 3901739 10.1002/ajim.4700080309

[B26] Sierra-Alvarez R Lettinga G The methanogenic toxicity of wood resin constituents Biological Wastes 1990 33 3 211 226 10.1016/0269-7483(90)90006-E

[B27] Taylor B List of Toxic woods to man 1990 June: American Woodturner

[B28] Woods B Calnan CD Toxic Woods Brit J Dermatol 1976 94 13 1 98 10.1111/j.1365-2133.1976.tb15776.x 132958

[B29] Koku JE Tree planting, local knowledge and species preference in the South Tongu District of Ghana: Some perspectives GeoJournal 2002 57 4 227 239

[B30] Grundy IM Campbell BM Balebereho S Cunliffe R Tafangenyasha C Fergusson R Parry D Availability and use of trees in Mutanda Resettlement Area Zimbabwe Forest Ecology and Management 1993 56 1 243 266 10.1016/0378-1127(93)90116-5

[B31] Pleydell G The tropical timbers of Ghana 1994 Ghana (TEDB): Timber Export Development Board

[B32] Foggie A Some ecological observations on a tropical forest type in the Gold Coast J Ecol 1947 34 1 88 106 10.2307/2256761

[B33] Bremananth R Nithya B Saipriya R Wood Species Recognition System World Academy of Science Engineering and Technology 2009 52 128 873 879

[B34] Oze G Nwanjo H Oze R Akubugwo E Orisakwe E Aka P Reproductive impairment associated with the ethanolic extract of alstonia boonei (de-wild) stem bark in male rats The Internet Journal of Laboratory Medicine 2008 3 1 10.5580/1e9c

[B35] Oze GO Nwanjo HU Onyeze GO Nephrotoxicity caused by the extract of alstonia boonei (de wild) stem bark in Guinea Pigs The Internet Journal of Nutrition and Wellness 2007 3 2 10.5580/398

[B36] Blay D The Distribution, Density, and Estimates of Carbon and Inorganic Nutrients in some Lesser-Used Species 1998 City Hotel, Kumasi, Ghana: ITTO/ FORIG/TEDB - VAPHU Conference

[B37] Adei E Nunoo L Yankey E Some Ghanaian Herbal Blood Tonics as Sources of Iron and other Trace Elements (Cu, Zn, Mn, Cd, Pb) J Sci Technol 2009 29 1 3

[B38] Falodun A Obasuyi O Phytochemical Screening and Evaluation of Stem Bark Extract of *Khaya senegalensis* (Meliaceae) on Methicillin Resistant *Staphyloccocus areus Canadian* Journal of Pure and Applied Sciences 2009 31 3 925 928

[B39] Adesogan EK Taylor DAH Limonoid extractives from Khaya ivorensis J Chem Soc (C) 1970 1710 1714 10.1039/J39700001710

[B40] Abdelgaleil SAM Hashinaga F Nakatani M Antifungal activity of limonoids from *Khaya ivorensis Pest Manag* Sci 2005 61 1 186 190 10.1002/ps.978 15619711

[B41] Australia New Zealand Food Authority Pyrrolizidine alkaloids in food: a toxicological review and risk assessment. Technical Report Series 2001. No 2 http://www.foodstandards.gov.au/_srcfiles/TR2.pdf

[B42] Gilbert RJ Watson HM Some laboratory experiments on various meat preparation surfaces with regard to surface contamination and cleaning J Food Technology 1971 6 1 163 170

[B43] Abrishami SH Tall BD Bruursema TJ Epstein PS Shah DB Bacteria adherence and viability on cutting board surfaces J Food Safety 1994 14 1 153 172

[B44] Gough NL Dodd CER The survival and disinfection of Salmonella typhimurium on chopping board surfaces of wood and plastic Food Control 1998 9 1 363 368

[B45] Milling A Kehr R Wulf A Smalla K The use of wood in practice–a hygienic risk? European Journal of Wood and Wood Products 2005 63 6 463 472 10.1007/s00107-005-0064-x

[B46] Schönwälder A Kehr R Wulf A Smalla K Wooden boards affecting the survival of bacteria? Holz Roh- Werkst 2002 60 4 249 257 10.1007/s00107-002-0300-6

[B47] Ak NO Cliver DO Kaspar CW Cutting boards of plastic and wood contaminated experimentally with bacteria J Food Protect 1994 57 1 16 22 10.4315/0362-028X-57.1.16 31113021

[B48] Boursillon D Riethmüller V The safety of wooden cutting boards: remobilization of bacteria from pine, beech, and polyethylene British Food Journal 2007 109 4 315 322 10.1108/00070700710736561

[B49] Carpentier B Sanitary quality of meat chopping board surfaces: a bibliographical study Food Microbiol 1997 14 1 31 37 10.1006/fmic.1996.0061

[B50] Dawson P Han I Cox C Simmons L Residence time and food contact time effects on transfer of Salmonella Typhimurium from tile, wood and carpet: testing the five-second rule J Appl Microbiol 2007 102 1 945 953 17381737 10.1111/j.1365-2672.2006.03171.x

[B51] Moore G Blair IS McDowell DA Recovery and transfer of Salmonella typhimurium from four different domestic food contact surfaces J Food Prot 2007 70 10 2273 2280 17969608 10.4315/0362-028x-70.10.2273

[B52] Valsanen OM Mentu J Salkinoja-Salonen MS Bacteria in food packaging and board J Appl Bacteriol 1991 71 1 130 133 1917722 10.1111/j.1365-2672.1991.tb02967.x

[B53] Castle L Migration from recycled paper and board to dry foods. Research into the factors involved, leading to practical avoidance and amelioration measures World Food Regulation Review 2004 13 1 11 12

[B54] Franz R Migration modelling from food-contact plastics into foodstuffs as a new tool for consumer exposure estimation Food Addit Contam 2005 22 10 920 937 10.1080/02652030500157700 16227176

[B55] Johns SM Jickelis SM Read WA Castle L Studies on functional barriers to migration. Migration of benzophenone and model ink components from cottonboard to food during frozen storage and microwave heating Packag Technol Sci 2000 13 3 99 104 10.1002/1099-1522(200005)13:3<99::AID-PTS499>3.0.CO;2-K

[B56] Mousavi SM Desoby S Hardy J Mathematical modeling of volatiles compounds into packaging food via packaging free space J Food Engineering 1998 36 1 453 472

[B57] Begley T Castle L Feigenbaum A Franz R Hinrichs K Lickly T Mercea P Milana M O’Brien A Rebre S Rijk R Piringer O Evaluation of migration models that might be used in support of regulations for food-contact plastics Food Addit Contam 2005 22 1 73 90 10.1080/02652030400028035 15895614

[B58] Tehrany EA Desobry S Partition coefficients in food/packaging systems: a review Food Addit Contam 2004 21 12 1186 1202 10.1080/02652030400019380 15799564

[B59] Kumar D Anti-inflammatory, analgesic, and antioxidant activities of methanolic wood extract of Pterocarpus santalinus L J Pharmacol Pharmacother 2011 2 3 200 202 10.4103/0976-500X.83293 21897722 PMC3157138

[B60] Mihara R Barry KM Mohammed CL Mitsunaga T Comparison of antifungal and antioxidant activities of *Acacia mangium and A. auriculiformis* heartwood extracts J Chemical Ecology 2005 31 4 789 804 10.1007/s10886-005-3544-x 16124251

[B61] Santana ALBD Maranhão CA Santos JC Cunha FM Conceição GM Bieber LW Nascimento MS Antitermitic activity of extractives from three Brazilian hardwoods against *Nasutitermes corniger* Int Biodeter Biodegr 2010 64 1 7 12 10.1016/j.ibiod.2009.07.009

[B62] Tung YT Hsu CA Chen CS Yang SC Huang CC Chang ST Phytochemicals from Acacia confusa heartwood extracts reduce serum uric acid levels in oxonate-induced mice: their potential use as xanthine oxidase inhibitors J Agric Food Chem 2010 58 18 9936 9941 10.1021/jf102689k 20806936

[B63] Välimaa AL Honkalampi-Hämäläinen U Pietarinen S Willför S Holmbom B von Wright A Antimicrobial and cytotoxic knotwood extracts and related pure compounds and their effects on food-associated microorganisms Int J Food Microbiol 2007 30 2 235 243 17188387 10.1016/j.ijfoodmicro.2006.10.031

[B64] Jayeola AA Aworinde DO Folorunso AE Use of Wood Characters in the Identifcation of Selected Timber Species in Nigeria Not Bot Hort Agrobot Cluj 2009 37 2 28 32

[B65] Khalid M Lee ELY Yusof R Nadaraj M Design of an intelligent wood species recognition system IJSSST 2008 9 3 1 5

[B66] Lawrence AH Barbour RJ Sutcliffe R Identification of wood species by ion mobility spectrometry Anal Chem 1991 63 13 1217 1221 10.1021/ac00013a007

[B67] Lhate I Cuvilas C Terziev N Jirjisb R Chemical composition of traditionally and lesser used wood species from Mozambique Wood Material Science and Engineering 2010 5 4 143 150 10.1080/17480272.2010.484867

[B68] Uçar G Balaban M The composition of volatile extractives from the wood of Juniperus excelsa, Juniperus foetidissima and Juniperus oxycedrus European J Wood and Wood Products 2002 60 5 356 362 10.1007/s00107-002-0316-y

[B69] Bertaud F Holmbom B Chemical composition of earlywood and latewood in Norway spruce heartwood, sapwood and transition zone wood Wood Sci Technol 2004 38 4 245 256

[B70] Charlet P Leno G Joseleau B Chareyre P Analysis of extractives from different wood species.4thCanadian Pulp and Paper Association, ISWPC 1997

[B71] Ostroukhova LA Babkin VA Malkov YA Babkin DV Onuchina NA Ivanova SZ Isolation of biologically active compounds from larch wood 1998

[B72] Redzynia I Ziółkowska NE Majzner WR Willför S Sjöholm R Eklund P Bujacz GD Structural investigation of biologically active phenolic compounds isolated from European tree species Molecules 2009 14 10 4147 4158 10.3390/molecules14104147 19924053 PMC6255330

[B73] Diserens JM Rapid determination of nineteen chlorophenols in wood, paper, cardboard, fruits, and fruit juices by gas chromatography/mass spectrometry J AOAC Int 2001 84 3 853 860 11417649

